# Sensitivity analysis of Wasserstein distributionally robust optimization problems

**DOI:** 10.1098/rspa.2021.0176

**Published:** 2021-12

**Authors:** Daniel Bartl, Samuel Drapeau, Jan Obłój, Johannes Wiesel

**Affiliations:** ^1^ Department of Mathematics, University of Vienna, Oskar-Morgenstern-Platz 1, 1090 Vienna, Austria; ^2^ School of Mathematical Sciences & Shanghai Advanced Institute of Finance, Shanghai Jiao Tong University, 211 West Huaihai Road, Shanghai 200030, People’s Republic of China; ^3^ Mathematical Institute, University of Oxford, Woodstock Road, Oxford OX2 6GG, UK; ^4^ Department of Statistics, Columbia University, 1255 Amsterdam Avenue, New York, NY 10027, USA

**Keywords:** robust stochastic optimization, sensitivity analysis, uncertainty quantification, non-parametric uncertainty, Wasserstein metric

## Abstract

We consider sensitivity of a generic stochastic optimization problem to model uncertainty. We take a non-parametric approach and capture model uncertainty using Wasserstein balls around the postulated model. We provide explicit formulae for the first-order correction to both the value function and the optimizer and further extend our results to optimization under linear constraints. We present applications to statistics, machine learning, mathematical finance and uncertainty quantification. In particular, we provide an explicit first-order approximation for square-root LASSO regression coefficients and deduce coefficient shrinkage compared to the ordinary least-squares regression. We consider robustness of call option pricing and deduce a new Black–Scholes sensitivity, a non-parametric version of the so-called Vega. We also compute sensitivities of optimized certainty equivalents in finance and propose measures to quantify robustness of neural networks to adversarial examples.

## 1. Introduction

We consider a generic stochastic optimization problem

1.1 $$\underset{a\in A}{\inf}\int_{S}f(x,a)\;\mu (\text{d}x),$$

where A is the set of actions or choices, f is the loss function and μ is a probability measure over the state space S. Such problems are found across the whole of applied mathematics. The measure μ is the crucial input and it could represent, for example, a dynamic model of the system, as is often the case in mathematical finance or mathematical biology, or the empirical measure of observed data points, or the training set, as is the case in statistics and machine learning applications. In virtually all the cases, there is a certain degree of uncertainty around the choice of μ coming from modelling choices and simplifications, incomplete information, data errors, finite sample error, etc. It is thus very important to understand the influence of changes in μ on (1.1), both on its value and on its optimizer. Often, the choice of μ is done in two stages: first a parametric family of models is adopted and then the values of the parameters are fixed. Sensitivity analysis of (1.1) with changing parameters is a classical topic explored in parametric programming and statistical inference, e.g. [1–3]. It also underscores a lot of progress in the field of uncertainty quantification, see [4]. Considering μ as an abstract parameter, the mathematical programming literature looked into qualitative and quantitative stability of (1.1). We refer to [5,6] and the references therein. When μ represents data samples, there has been a considerable interest in the optimization community in designing algorithms which are robust and, in particular, do not require excessive hypertuning, see [7] and the references therein.

A more systematic approach to model uncertainty in (1.1) is offered by the distributionally robust optimization problem

1.2 $$V(\delta ):=\underset{a\in A}{\inf}V(\delta ,a):=\underset{a\in A}{\inf}\underset{\nu \in B_{\delta }(\mu )}{\sup}\int_{S}f(x,a)\;\nu (\text{d}x),$$

where $B_{\delta }(\mu )$ is a ball of radius δ around μ in the space of probability measures, as specified below. Such problems greatly generalize more classical robust optimization and have been studied extensively in operations research and machine learning in particular; we refer the reader to [8] and the references therein. Our goal in this paper is to understand the behaviour of these problems for small δ. Our main results compute first-order behaviour of V(δ) and its optimizer for small δ. This offers a measure of sensitivity to errors in model choice and/or specification as well as points in the abstract direction, in the space of models, in which the change is most pronounced. We use examples to show that our results can be applied across a wide spectrum of science.

This paper is organized as follows. We first present the main results and then, in §3, explore their applications. Further discussion of our results and the related literature is found in §4, which is then followed by the proofs. The online appendix [9] contains many supplementary results and remarks, as well as some more technical arguments from the proofs.

## 2. Main results

Take d,k∈N, endow $R^{d}$ with the Euclidean norm |⋅| and write $\Gamma^{o}$ for the interior of a set Γ. Assume that S is a closed convex subset of $R^{d}$. Let P(S) denote the set of all (Borel) probability measures on S. Further fix a seminorm ||⋅|| on $R^{d}$ and denote by $||\cdot |{|}_{*}$ its (extended) dual norm, i.e. $||y|{|}_{*}:=\sup\{\langle x,y\rangle :||x||\leq 1\}$. In particular, for ||⋅||=|⋅| we also have $||\cdot |{|}_{*}=|\cdot |$. For μ,ν∈P(S), we define the p-Wasserstein distance as

$$W_{p}(\mu ,\nu )=\inf{\left\{\int_{S\times S}||x-y|{|}_{*}^{p}\;\pi (\text{d}x,\text{d}y):\pi \in \mathrm{Cpl}(\mu ,\nu )\right\}}^{1/p},$$

where Cpl(μ,ν) is the set of all probability measures π on S×S with first marginal $\pi_{1}:=\pi (\cdot \times S)=\mu$ and second marginal $\pi_{2}:=\pi (S\times \cdot )=\nu$. Denote the Wasserstein ball

$$B_{\delta }(\mu )=\{\nu \in P(S):W_{p}(\mu ,\nu )\leq \delta \},$$

of size δ≥0 around μ. Note that, taking a suitable probability space (Ω,F,P) and a random variable X∼μ, we have the following probabilistic representation of V(δ,a):

$$\underset{\nu \in B_{\delta }(\mu )}{\sup}\int_{S}f(x,a)\;\nu (\text{d}x)=\underset{Z}{\sup}E_{P}[f(X+Z,a)],$$

where the supremum is taken over all Z satisfying X+Z∈S almost surely and $E_{P}[||Z|{|}_{*}^{p}]\leq \delta^{p}$. Wasserstein distances and optimal transport techniques have proved to be powerful and versatile tools in a multitude of applications, from economics [10,11] to image recognition [12]. The idea to use Wasserstein balls to represent model uncertainty was pioneered in [13] in the context of investment problems. When sampling from a measure with a finite pth moment, the measures converge to the true distribution and Wasserstein balls around the empirical measures have the interpretation of confidence sets, see [14]. In this set-up, the radius δ can then be chosen as a function of a given confidence level α and the sample size N. This yields finite sample guarantees and asymptotic consistency, see [15,16], and justifies the use of the Wasserstein metric to capture model uncertainty. The value V(δ,a) in (1.2) has a dual representation, see [17,18], which has led to significant new developments in distributionally robust optimization, e.g.[15,19–21].

Naturally, other choices for the distance on the space of measures are also possible: such as the Kullblack–Leibler divergence, see [22] for general sensitivity results and [23] for applications in portfolio optimization, or the Hellinger distance, see [24] for a statistical robustness analysis. We refer to §4 for a more detailed analysis of the state of the art in these fields. Both of these approaches have good analytic properties and often lead to theoretically appealing closed-form solutions. However, they are also very restrictive since any measure in the neighbourhood of μ has to be absolutely continuous with respect to μ. In particular, if μ is the empirical measure of N observations then measures in its neighbourhood have to be supported on those fixed N points. To obtain meaningful results, it is thus necessary to impose additional structural assumptions, which are often hard to justify solely on the basis of the data at hand and, equally importantly, create another layer of model uncertainty themselves. We refer to [17, sec. 1.1] for further discussion of potential issues with ϕ-divergences. The Wasserstein distance, while harder to handle analytically, is more versatile and does not require any such additional assumptions.

Throughout the paper, we take the convention that continuity and closure are understood w.r.t. |⋅|. We assume that $A\subset R^{k}$ is convex and closed and that the seminorm ||⋅|| is strictly convex in the sense that for two elements $x,y\in R^{d}$ with ||x||=||y||=1 and ||x−y||≠0, we have $||\frac{1}{2}x+\frac{1}{2}y||<1$ (note that this is satisfied for every $l^{s}$-norm $|x{|}_{s}:={\left(\sum_{i=1}^{d}|x_{i}{|}^{s}\right)}^{1/s}$ for s>1). We fix p∈(1,∞), let q:=p/(p−1) so that 1/p+1/q=1, and fix μ∈P(S) such that the boundary of $S\subset R^{d}$ has μ–zero measure and $\int_{S}|x{|}^{p}\;\mu (\text{d}x)<\infty$. Denote by $A_{\delta }^{⋆}$ the set of optimizers for V(δ) in (1.2).

**Assumption 2.1.**

The loss function f:S×A→R satisfies

— x↦f(x,a) is differentiable on $S^{o}$ for every a∈A. Moreover, $\left(x,a)\mapsto \nabla_{x}f(x,a\right)$ is continuous and for every r>0 there is c>0 such that $|\nabla_{x}f(x,a)|\leq c(1+|x{|}^{p-1})$ for all x∈S and a∈A with |a|≤r.
— For all δ≥0 sufficiently small, we have $A_{\delta }^{⋆}\neq \emptyset$ and for every sequence ${\left(\delta_{n}\right)}_{n\in N}$ such that $\lim_{n\to \infty }\delta_{n}=0$ and ${\left(a_{n}^{⋆}\right)}_{n\in N}$ such that $a_{n}^{⋆}\in A_{\delta_{n}}^{⋆}$ for all n∈N there is a subsequence which converges to some $a^{⋆}\in A_{0}^{⋆}$.

The above assumption is not restrictive: the first part merely ensures existence of $||\nabla_{x}f(\cdot ,a^{⋆})|{|}_{L^{q}(\mu )}$, while the second part is satisfied as soon as either A is compact or V(0,⋅) is coercive, which is the case in most examples of interest; see [9, lemma 7.15] for further comments.

**Theorem 2.2.**

*If assumption 2.1 holds then* $V^{\prime }(0)$ *is given by*

$$\Upsilon :=\lim_{\delta \to 0}\frac{V(\delta )-V(0)}{\delta }=\underset{a^{⋆}\in A_{0}^{⋆}}{\inf}{\left(\int_{S}||\nabla_{x}f(x,a^{⋆})|{|}^{q}\;\mu (\text{d}x)\right)}^{1/q}.$$

**Remark.**

Inspecting the proof, defining

$$\tilde{V}(\delta )=\underset{a^{⋆}\in A_{0}^{⋆}}{\inf}\underset{\nu \in B_{\delta }(\mu )}{\sup}\int_{S}f(x,a^{⋆})\;\nu (\text{d}x)$$

we obtain ${\tilde{V}}^{\prime }(0)=V^{\prime }(0)$. This means that for small δ>0 there is no first-order gain from optimizing over all a∈A in the definition of V(δ) when compared with restricting simply to $a^{⋆}\in A_{0}^{⋆}$, as in $\tilde{V}(\delta )$.

The above result naturally extends to computing sensitivities of robust problems, i.e. $V^{\prime }(r)$, see [9, corollary 7.5], as well as to the case of stochastic optimization under linear constraints, see [9, theorem 7.7]. We recall that $V(0,a)=\int_{S}f(x,a)\;\mu (\text{d}x)$.

**Assumption 2.3.**

Suppose the f is twice continuously differentiable, $a^{⋆}\in A_{0}^{⋆}\cap A^{o}$ and

— $\sum_{i=1}^{k}|\nabla_{a_{i}}\nabla_{x}f(x,a)|\leq c(1+|x{|}^{p-1-\varepsilon })$ for some ε>0, c>0, all x∈S and all a close to $a^{⋆}$.
— The function a↦V(0,a) is twice continuously differentiable in the neighbourhood of $a^{⋆}$ and the matrix $\nabla_{a}^{2}V(0,a^{⋆})$ is invertible.

**Theorem 2.4.**

*Suppose* $a^{⋆}\in A_{0}^{⋆}$ *and* $a_{\delta }^{⋆}\in A_{\delta }^{⋆}$ *such that* $a_{\delta }^{⋆}\to a^{⋆}$ *as* δ→0 *and assumptions 2.1 and 2.3 are satisfied. If* $\nabla_{x}f(x,a^{⋆})\neq 0$ μ-*a.e. or if* $\nabla_{x}\nabla_{a}f(x,a^{⋆})=0$ μ-*a.e., then*

$$\begin{array}{rl} ℶ & \;:=\lim_{\delta \to 0}\frac{a_{\delta }^{⋆}-a^{⋆}}{\delta }=-{\left(\int_{S}||\nabla_{x}f(x,a^{⋆})|{|}^{q}\;\mu (\text{d}x)\right)}^{(1/q)-1}\\ & \;\;\cdot (\nabla_{a}^{2}V{\left(0,a^{⋆})\right)}^{-1}\int_{S}\frac{\nabla_{x}\nabla_{a}f(x,a^{⋆})\;h(\nabla_{x}f(x,a^{⋆}))}{||\nabla_{x}f(x,a^{⋆})|{|}^{1-q}}\;\mu (\text{d}x), \end{array}$$

*where* $h:R^{d}\setminus \{0\}\to \{x\in R^{d}\;:\;||x|{|}_{*}=1\}$ *is the unique function satisfying* ⟨⋅,h(⋅)⟩=||⋅||, *see* [9, *Lemma* 6.2]. *In particular*, h(⋅)=⋅/|⋅| *if* ||⋅||=|⋅|.

Above and throughout the convention is that $\nabla_{x}f(x,a)\in R^{d\times 1},$ $\nabla_{a_{i}}\nabla_{x}f(x,a)\in R^{d\times 1}$, $\nabla_{a}f(x,a)\in R^{k\times 1}$, $\nabla_{x}\nabla_{a}f(x,a)\in R^{k\times d}$ and 0/0=0. The assumed existence and convergence of optimizers holds, e.g. with suitable convexity of f in a; see [9, lemma 7.14] for a worked out setting. In line with the financial economics practice, we gave our sensitivities letter symbols, Υ and ℶ, loosely motivated by $\Upsilon \pi \overset{´}{o}\delta \varepsilon \iota \gamma \mu \alpha$, the Greek for *Model*, and 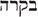, the Hebrew for *control*.

## 3. Applications

We now illustrate the universality of theorems 2.2 and 2.4 by considering their applications in a number of different fields. Unless otherwise stated, $S=R^{d}$, $A=R^{k}$ and ∫ means $\int_{S}$.

### (a) Financial economics

We start with the simple example of risk-neutral pricing of a call option written on an underlying asset ${\left(S_{t}\right)}_{t\leq T}$. Here, T,K>0 are the maturity and the strike, respectively, $f(x,a)={\left(S_{0}x-K\right)}^{+}$ and μ is the distribution of $S_{T}/S_{0}$. We set interest rates and dividends to zero for simplicity. In [25], the model μ is a lognormal distribution, i.e. $\log(S_{T}/S_{0})∼N(-\sigma^{2}T/2,\sigma^{2}T)$ is Gaussian with mean $-\sigma^{2}T/2$ and variance $\sigma^{2}T$. In this case, V(0) is given by the celebrated Black–Scholes formula. Note that this example is particularly simple since f is independent of a. However, to ensure risk-neutral pricing, we have to impose a linear constraint on the measures in $B_{\delta }(\mu )$, giving

3.1 $$\underset{\nu \in B_{\delta }(\mu ):\int_{\;}^{\;}x\nu (\text{d}x)=1}{\sup}\int_{\;}^{\;}{\left(S_{0}x-K\right)}^{+}\nu (\text{d}x).$$

To compute its sensitivity we encode the constraint using a Lagrangian and apply theorem 2.2, see [9, remark 7.3, theorem 7.7]. For p=2, letting $k=K/S_{0}$ and $\mu_{k}=\mu ([k,\infty ))$, the resulting formula, see [9, example 7.10], is given by

$$\Upsilon =S_{0}\sqrt{\int ({\text{1}}_{x\geq k}-\mu_{k}{)}^{2}\mu (\text{d}x)}=S_{0}\sqrt{\mu_{k}(1-\mu_{k})}.$$

**Figure 1.**
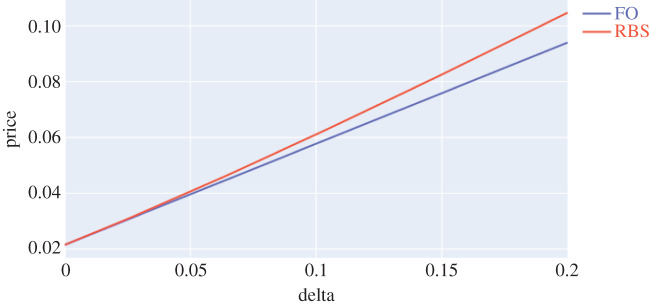
DRO value RBS(δ) versus the first order (FO) approximation RBS(0)+Υδ, $S_{0}=T=1$, K=1.2, σ=0.2. (Online version in colour.)

**Figure 2.**
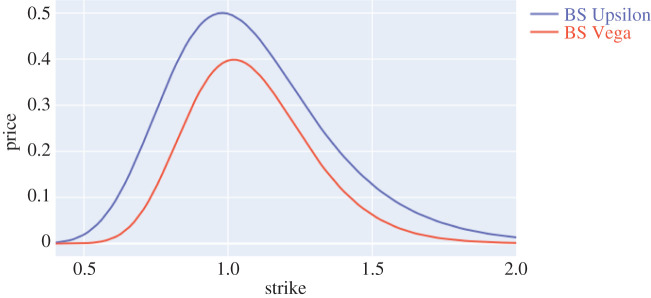
Black–Scholes model: Υ versus V, $S_{0}=T=1$, σ=0.2. (Online version in colour.)

Let us specialize to the lognormal distribution of the Black–Scholes model above and denote the quantity in (3.1) as RBS(δ). It may be computed exactly using methods from [26]. Figure 1 compares the exact value and the first-order approximation. We have $\Upsilon =S_{0}\sqrt{\Phi (d_{-})(1-\Phi (d_{-}))}$, where $d_{-}=\log(S_{0}/K)-\sigma^{2}T/2/\sigma \sqrt{T}$ and Φ is the cdf of N(0,1) distribution. It is also insightful to compare Υ with a parametric sensitivity. If instead of Wasserstein balls, we consider $\left\{N(-{\tilde{\sigma }}^{2}T/2,{\tilde{\sigma }}^{2}T):|\sigma -\tilde{\sigma }|\leq \delta \right\}$ the resulting sensitivity is known as the Black–Scholes Vega and given by $V=S_{0}\Phi^{\prime }(d_{-}+\sigma \sqrt{T})$. We plot the two sensitivities in figure 2. It is remarkable how, for the range of strikes of interest, the non-parametric model sensitivity Υ traces out the usual shape of V but shifted upwards to account for the idiosyncratic risk of departure from the lognormal family. More generally, given a book of options with payoff $f=f^{+}-f^{-}$ at time T, with $f^{+}$, $f^{-}\geq 0$, we could say that the book is Υ-neutral if the sensitivity Υ was the same for $f^{+}$ and for $f^{-}$. In analogy to Delta-Vega hedging standard, one could develop a non-parametric model-agnostic Delta-Upsilon hedging. We believe these ideas offer potential for exciting industrial applications and we leave them to further research.

We turn now to the classical notion of the optimized certainty equivalent (OCE) of [27]. It is a decision theoretic criterion designed to split a liability between today's and tomorrow’s payments. It is also a convex risk measure in the sense of [28] and covers many of the popular risk measures such as expected shortfall or entropic risk, see [29]. We fix a convex monotone function l:R→R which is bounded from below and $g:R^{d}\to R$. Here, g represents the payoff of a financial position and l is the negative of a utility function, or a loss function. We take ||⋅||=|⋅| and refer to [9, lemma 7.14] for generic sufficient conditions for assumptions 2.1 and 2.3 to hold in this setup. The OCE corresponds to V in (1.1) for f(x,a)=l(g(x)−a)+a and A=R, $S=R^{d}$. Theorems 2.2 and 2.4 yield the sensitivities

$$\begin{array}{rl} & \Upsilon =\underset{a^{⋆}\in A_{0}^{⋆}}{\inf}{\left(\int |l^{\prime }(g(x)-a^{⋆})\nabla g(x){|}^{q}\;\mu (\text{d}x)\right)}^{1/q},\\ & ℶ=(\int |l^{\prime }(g(x)-a^{⋆})\;\nabla g(x){|}^{2}\;\mu (\text{d}z){)}^{-1/2}\cdot \frac{\int l^{\prime \prime }(g(x)-a^{⋆})\;l^{\prime }(g(x)-a^{⋆})\;{\left(\nabla g(x)\right)}^{2}\;\mu (\text{d}x)}{\int l^{\prime \prime }(g(x)-a^{⋆})\;\mu (\text{d}x)}, \end{array}$$

where, for simplicity, we took p=q=2 for the latter.

A related problem considers hedging strategies which minimize the expected loss of the hedged position, i.e. $f(x,a)=l(g(x)+\langle a,x-x_{0}\rangle )$, where $A=R^{k}$ and $\left(x_{0},x\right)$ represent today's and tomorrow’s traded prices. We compute Υ as

$$\underset{a^{⋆}\in A_{0}^{⋆}}{\inf}{\left(\int |l^{\prime }(g(x)+\langle a^{⋆},x-x_{0}\rangle )(\nabla g(x)+a^{⋆}){|}^{q}\;\mu (\text{d}x)\right)}^{1/q}.$$

Furthermore we can combine loss minimization with OCE and consider $a=(H,m)\in R^{k}\times R$, $f(x,(h,m))=l(g(x)+\langle H,x-x_{0}\rangle +m)-m$. This gives $V^{\prime }(0)$ as the infimum over $(H^{⋆},m^{⋆})\in A_{0}^{⋆}$ of

$${\left(\int |l^{\prime }(g(x)+\langle H^{⋆},x-x_{0}\rangle +m^{⋆})(\nabla g(x)+H^{⋆}){|}^{q}\;\mu (\text{d}x)\right)}^{1/q}.$$

The above formulae capture non-parametric sensitivity to model uncertainty for examples of key risk measurements in financial economics. To the best of our knowledge, this has not been achieved before.

Finally, we consider briefly the classical mean-variance optimization of [30]. Here μ represents the loss distribution across the assets and $a\in R^{d}$, $\sum_{i=1}^{d}a_{i}=1$ are the relative investment weights. The original problem is to minimize the sum of the expectation and γ standard deviations of returns ⟨a,X⟩, with X∼μ. Using the ideas in [31, Example 2] and considering measures on $R^{d}\times R^{d}$, we can recast the problem as (1.1). While [31] focused on the asymptotic regime δ→∞, their non-asymptotic statements are related to our theorem 2.2 and either result could be used here to obtain that $V(\delta )\approx V(0)+\sqrt{1-\gamma^{2}}\delta$ for small δ.

### (b) Neural networks

We specialize now to quantifying robustness of neural networks (NN) to adversarial examples. This has been an important topic in machine learning since [32] observed that NN consistently misclassify inputs formed by applying small worst-case perturbations to a dataset. This produced a number of works offering either explanations for these effects or algorithms to create such adversarial examples, e.g. [33–39] to name just a few. The main focus of research works in this area, see [40], has been on faster algorithms for finding adversarial examples, typically leading to an overfit to these examples without any significant generalization properties. The viewpoint has been mainly pointwise, e.g. [32], with some generalizations to probabilistic robustness, e.g. [39].

In contrast, we propose a simple metric for measuring robustness of NN which is independent of the architecture employed and the algorithms for identifying adversarial examples. In fact, theorem 2.2 offers a simple and intuitive way to formalize robustness of NN: for simplicity consider a 1-layer neural network trained on a given distribution μ of pairs (x,y), i.e. $\left(A_{1}^{⋆},A_{2}^{⋆},b_{1}^{⋆},b_{2}^{⋆}\right)$ solve

$$\inf\int |y-((A_{2}(\cdot )+b_{2})\circ \sigma \circ (A_{1}(\cdot )+b_{1}))(x){|}^{p}\;\mu (\text{d}x,\text{d}y),$$

where the inf is taken over $a=(A_{1},A_{2},b_{1},b_{2})\in A=R^{k\times d}\times R^{d\times k}\times R^{k}\times R^{d}$, for a given activation function σ:R→R, where the composition above is understood componentwise. Set $f(x,y;A,b):=|y-(A_{2}(\cdot )+b_{2})\circ \sigma \circ (A_{1}(\cdot )+b_{1})(x){|}^{p}$. Data perturbations are captured by $\nu \in B_{\delta }^{p}(\mu )$ and (1.2) offers a robust training procedure. The first-order quantification of the NN sensitivity to adversarial data is then given by

$${\left(\int |\nabla f(x,y;A^{⋆},b^{⋆}){|}^{q}\;\mu (\text{d}x,\text{d}y)\right)}^{1/q}.$$

A similar viewpoint, capturing robustness to adversarial examples through the optimal transport lens, has been recently adopted by other authors. The dual formulation of (1.2) was used by [21] to reduce the training of neural networks to tractable linear programs. [41] modified (1.2) to consider a penalized problem $\underset{a\in A}{\inf}\underset{\nu \in P(S)}{\sup}\int_{S}f(x,a)\;\nu (\text{d}x)-\gamma W_{p}(\mu ,\nu )$ to propose new stochastic gradient descent algorithms with inbuilt robustness to adversarial data.

### (c) Uncertainty quantification

In the context of UQ, the measure μ represents input parameters of a (possibly complicated) operation G in a physical, engineering or economic system. We consider the so-called *reliability* or *certification problem*: for a given set E of undesirable outcomes, one wants to control $\underset{\nu \in P}{\sup}\nu (G(x)\in E)$, for a set of probability measures P. The distributionally robust adversarial classification problem considered recently by [42] is also of this form, with Wasserstein balls P around an empirical measure of N samples. Using the dual formulation of [18], they linked the problem to minimization of the conditional value-at-risk and proposed a reformulation, and numerical methods, in the case of linear classification. We propose instead a regularized version of the problem and look for

$$\delta (\alpha ):=\sup\left\{\delta \geq 0:\;\underset{\nu \in B_{\delta }(\mu )}{\inf}\int \;\text{d}(G(x),E)\;\nu (\text{d}x)\geq \alpha \right\},$$

for a given safety level α. We thus consider the average distance to the undesirable set, $d(G(x),E):=\underset{e\in E}{\inf}|G(x)-e|$, and not just its probability. The quantity δ(α) could then be used to quantify the implicit uncertainty of the certification problem, where higher δ corresponds to less uncertainty. Taking statistical confidence bounds of the empirical measure in Wasserstein distance into account, see [14], δ would then determine the minimum number of samples needed to estimate the empirical measure.

Assume that E is convex. Then x↦d(x,E) differentiable everywhere except at the boundary of E with $\nabla_{x}d(x,E)=0$ for $x\in E^{o}$ and $|\nabla_{x}d(x,E)|=1$ for all $x\in {\bar{E}}^{c}$. Furthermore, assume μ is absolutely continuous w.r.t. Lebesgue measure on S. Theorem 2.2, using [9, remark 7.3], gives a first-order expansion for the above problem:

$$\begin{array}{r} \underset{\nu \in B_{\delta }(\mu )}{\inf}\int \;\text{d}(G(x),E)\;\nu (\text{d}x)=\int \;\text{d}(G(x),E)\;\mu (\text{d}x)-{\left(\int |\nabla_{x}d(G(x),E)\nabla_{x}G(x){|}^{q}\;\mu (\text{d}x)\right)}^{1/q}\delta +o(\delta ). \end{array}$$

In the special case $\nabla_{x}G(x)=cI$ this simplifies to

$$\int d(G(x),E)\;\mu (\text{d}x)-c{\left(\mu (G(x)\notin E)\right)}^{1/q}\delta +o(\delta ),$$

and the minimal measure ν pushes every point G(x) not contained in E in the direction of the orthogonal projection. This recovers the intuition of [43, theorem 1], which in turn relies on [17, corollary 2, example 7]. Note however that our result holds for general measures μ. We also note that such an approximation could provide an ansatz for dimension reduction, by identifying the dimensions for which the partial derivatives are negligible and then projecting G on to the corresponding lower-dimensional subspace (thus providing a simpler surrogate for G). This would be an alternative to a basis expansion (e.g. in orthogonal polynomials) used in UQ and would exploit the interplay between the properties of G and μ simultaneously.

### (d) Statistics

We discuss two applications of our results in the realm of statistics. We start by highlighting the link between our results and the so-called *influence curves* (IC) in robust statistics. For a functional μ↦T(μ) its IC is defined as

$$\mathrm{IC}(y)=\lim_{t\to 0}\frac{T(t\delta_{y}+(1-t)\mu )-T(\mu )}{t}.$$

Computing the IC, if it exists, is in general hard and closed form solutions may be unachievable. However, for the so-called M-estimators, defined as optimizers for V(0),

$$T(\mu ):={\mathrm{argmin}}_{a}\int f(x,a)\mu (\text{d}x),$$

for some f (e.g. f(x,a)=|x−a| for the median), we have

$$\mathrm{IC}(y)=\frac{\nabla_{a}f(y,T(\mu ))}{-\int \nabla_{a}^{2}f(s,T(\mu ))\;\mu (\text{d}s)},$$

under suitable assumptions on f, see [44, section 3.2.1]. In comparison, writing $T^{\delta }$ for the optimizer for V(δ), theorem 2.4 yields

3.2 $$\lim_{\delta \to 0}\frac{T^{\delta }-T(\mu )}{\delta }=\frac{\int \nabla_{x}\nabla_{a}f(x,T(\mu ))\nabla_{x}f(x,T(\mu ))\;\mu (\text{d}x)}{-\int \nabla_{a}^{2}f(s,T(\mu ))\;\mu (\text{d}s)},$$

under assumption 2.3 and normalization $||\nabla_{x}f(x,T(\mu ))|{|}_{L^{p}(\mu )}=1$. To investigate the connection let us Taylor-expand IC(y) around x to obtain

$$\mathrm{IC}(y)-\mathrm{IC}(x)=\frac{\nabla_{a}\nabla_{x}f(x,T(\mu ))}{-\int \nabla_{a}^{2}f(s,T(\mu ))\;\mu (\text{d}s)}(y-x).$$

Choosing $y=x+\delta \nabla f_{x}(x,T(\mu ))$ and integrating both sides over μ and dividing by δ, we obtain the asymptotic equality

$$\int \frac{\mathrm{IC}(x+\delta \nabla_{x}f(x,T(\mu )))-\mathrm{IC}(x)}{\delta }\;\mu (\text{d}x)\approx \frac{T^{\delta }-T(\mu )}{\delta },$$

for δ→0 by (3.2). We conclude that considering the average directional derivative of IC in the direction of $\nabla f_{x}(x,T(\mu ))$ gives our first-order sensitivity. For an interesting conjecture regarding the comparison of influence functions and sensitivities in KL-divergence, we refer to [45, Section 7.3] and [22, Section 3.4.2].

Our second application in statistics exploits the representation of the LASSO/Ridge regressions as robust versions of the standard linear regression. We consider $A=R^{k}$ and $S=R^{k+1}$. If instead of the Euclidean metric we take $||(x,y)|{|}_{*}=|x{|}_{r}1_{\left\{y=0\right\}}+\infty 1_{\left\{y\neq 0\right\}}$, for some r>1 and $(x,y)\in R^{k}\times R$, in the definition of the Wasserstein distance, then [19] showed that

3.3 $$\begin{array}{r} \underset{a\in R^{k}}{\inf}\underset{\nu \in B_{\delta }(\mu )}{\sup}\int {\left(y-\langle x,a\rangle \right)}^{2}\;\nu (\text{d}x,\text{d}y)=\underset{a\in R^{k}}{\inf}{\left(\sqrt{\int {\left(y-\langle a,x\rangle \right)}^{2}\;\mu (\text{d}x,\text{d}y)}+\delta |a{|}_{s}\right)}^{2} \end{array}$$

holds, where 1/r+1/s=1. The δ=0 case is the ordinary least-squares regression. For δ>0, the r.h.s. for s=2 is directly related to the Ridge regression, while the limiting case s=1 is called the square-root LASSO regression, a regularized variant of linear regression well known for its good empirical performance. Closed-form solutions to (3.3) do not exist in general and it is a common practice to use numerical routines to solve it approximately. Theorem 2.4 offers instead an explicit first-order approximation of $a_{\delta }^{⋆}$ for small δ. We denote by $a^{⋆}$ the ordinary least-squares estimator and by I the k×k identity matrix. Note that the first-order condition on $a^{⋆}$ implies that $\int (y-\langle a^{⋆},x\rangle )x_{i}\mu (\text{d}x,\text{d}y)=0$ for all 1≤i≤k. In particular, $V(0)=\int (y^{2}-\langle a^{⋆},x\rangle y)\mu (\text{d}x,\text{d}y)$ and $a^{⋆}=D^{-1}\int yx\mu (\text{d}x,\text{d}y)$, where we assume the system is overdetermined so that $D=\int xx^{T}\;\mu (\text{d}x,\text{d}y)$ is invertible. A direct computation, see [9, example 8.2], yields

3.4 $$a_{\delta }^{⋆}\approx \;a^{⋆}-\sqrt{V(0)}D^{-1}\;h(a^{⋆})\delta .$$

For s=2, $h(a^{⋆})=a^{⋆}/|a^{⋆}{|}_{2}$ and for s=1, $h(a^{⋆})=\text{sign}(a^{⋆})$ and hence^1^ $a_{\delta }^{⋆}$ is approximately

**Figure 3.**
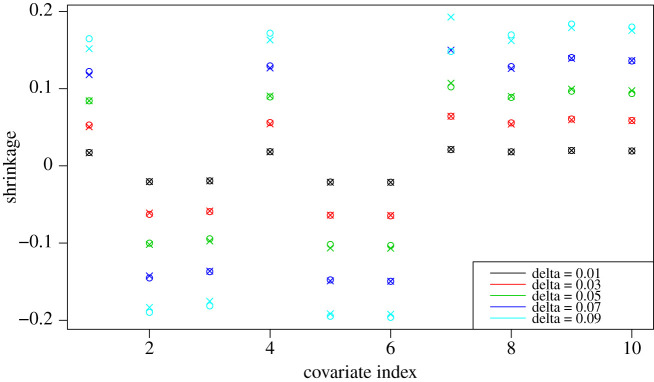
Square-root LASSO parameter shrinkage $a_{\delta }^{⋆}-a_{0}^{⋆}$: exact (o) and the first-order approximation (*x*) in (3.5). 2000 observations generated according to $Y=1.5X_{1}-3X_{2}-2X_{3}+0.3X_{4}-0.5X_{5}-0.7X_{6}+0.2X_{7}+0.5X_{8}+1.2X_{9}+0.8X_{10}+\varepsilon$ with all $X_{i}$, ε i.i.d. N(0,1). (Online version in colour.)

3.5 $$\left(1-\frac{\sqrt{V(0)}}{|a^{⋆}{|}_{2}}D^{-1}\delta \right)a^{⋆}\;\text{and}\;a^{⋆}-\sqrt{V(0)}D^{-1}\text{sign}(a^{⋆})\delta ,$$

respectively. This corresponds to parameter shrinkage: proportional for square-root Ridge and a shift towards zero for square-root LASSO. To the best of our knowledge, these are first such results and we stress that our formulae are valid in a general context and, in particular, parameter shrinkage depends on the direction through the $D^{-1}$ factor. Figure 3 compares the first-order approximation with the actual results and shows a remarkable fit. Furthermore, our results agree with what is known in the canonical test case for the (standard) Ridge and LASSO, see [46]. When $\mu =\mu_{N}$ is the empirical measure of N i.i.d. observations, the data are centred and the covariates are orthogonal, i.e. D=(1/N)I. In that case, (3.5) simplifies to

$$\left(1-\delta \sqrt{N\left(\frac{1}{R^{2}}-1\right)}\right)a^{⋆}\;\text{and}\;a^{⋆}-\sqrt{N}\;|y|\;\sqrt{1-R^{2}}\;\text{sign}(a^{⋆})\delta ,$$

where $R^{2}$ is the usual coefficient of determination.

The case of $\mu_{N}$ is naturally of particular importance in statistics and data science and we continue to consider it in the next subsection. In particular, we characterize the asymptotic distribution of $\sqrt{N}(a_{1/\sqrt{N}}^{⋆}-a^{⋆})$, where $a_{\delta }^{⋆}\in A_{\delta }^{⋆}(\mu_{N})$ and $a^{⋆}\in A_{0}^{⋆}(\mu_{\infty })$ is the optimizer of the non-robust problem for the data-generating measure. This recovers the central limit theorem of [47], a link we explain further in §4b.

### (e) Out-of-sample error

A benchmark of paramount importance in optimization is the so-called *out-of-sample error*, also known as the *prediction error* in statistical learning. Consider the setup above when $\mu_{N}$ is the empirical measure of N i.i.d. observations sampled from the ‘true’ distribution $\mu =\mu_{\infty }$ and take, for simplicity, $||\cdot ||=|\cdot {|}_{s}$, with s>1. Our aim is to compute the optimal $a^{⋆}$ which solves the original problem (1.1). However, we only have access to the training set, encoded via $\mu_{N}$. Suppose we solve the distributionally robust optimization problem (1.2) for $\mu_{N}$ and denote the robust optimizer $a_{\delta }^{⋆,N}$. Then the *out-of-sample error*

$$V(0,a_{\delta }^{⋆,N})-V(0,a^{⋆})=\int f(x,a_{\delta }^{⋆,N})\;\mu (\text{d}x)-\int f(x,a^{⋆})\;\mu (\text{d}x)$$

quantifies the error from using $a_{\delta }^{⋆,N}$ as opposed to the true optimizer $a^{⋆}$.

While this expression seems to be hard to compute explicitly for finite samples, theorem 2.4 offers a way to find the asymptotic distribution of a (suitably rescaled version of) the out-of-sample error. We suppose the assumptions in theorem 2.4 are satisfied and note that the first order condition for $a^{⋆}$ gives $\nabla_{a}V(0,a^{⋆})=0$. Then, a second-order Taylor expansion gives

3.6 $$V(0,a_{\delta }^{⋆,N})-V(0,a^{⋆})=\frac{1}{2}{\left(a_{\delta }^{⋆,N}-a^{⋆}\right)}^{T}\nabla_{a}^{2}V(0,\tilde{a})(a_{\delta }^{⋆,N}-a^{⋆}),$$

for some $\tilde{a}$ (coordinate-wise) between $a^{⋆}$ and $a_{\delta }^{⋆,N}$. Now we write

$$a_{\delta }^{⋆,N}-a^{⋆}=a_{\delta }^{⋆,N}-a^{⋆,N}+a^{⋆,N}-a^{⋆},$$

where we define $a^{⋆,N}$ as the optimizer of the non-robust problem (1.1) with μ replaced by $\mu_{N}$. In particular, the δ-method for M-estimators implies that

3.7 $$\sqrt{N}(a^{⋆,N}-a^{⋆})\Rightarrow {\left(\nabla_{a}^{2}V(0,a^{⋆})\right)}^{-1}H,$$

where $H∼N(0,\int {\left(\nabla_{a}f(x,a^{⋆})\right)}^{T}\nabla_{a}f(x,a^{⋆})\;\mu (\text{d}x))$ and ⇒ denotes the convergence in distribution. On the other hand, for a fixed N∈N, theorem 2.4 applied to $\mu_{N}$ yields

3.8 $$\begin{array}{rl} a_{\delta }^{⋆,N}-a^{⋆,N} & \;=-{\left(\int |\nabla_{x}f(x,a^{⋆,N}){|}_{s}^{q}\;\mu_{N}(\text{d}x)\right)}^{(1/q)-1}\cdot {\left(\int \nabla_{a}^{2}f(x,a^{⋆,N})\;\mu_{N}(\text{d}x)\right)}^{-1}\\ & \;\;\cdot \int \frac{\nabla_{x}\nabla_{a}f(x,a^{⋆,N})\;h(\nabla_{x}f(x,a^{⋆,N}))}{|\nabla_{x}f(x,a^{⋆,N}){|}_{s}^{1-q}}\;\mu_{N}(\text{d}x)\cdot \delta +o(\delta ) \end{array}$$

3.9 $$\begin{array}{rl} & \;=-{\left((\nabla_{a}^{2}V(0,a^{⋆})\right)}^{-1}\Theta +\Delta_{N})\cdot \delta +o(\delta ), \end{array}$$

where

$$\begin{array}{rl} \Theta & \;:={\left(\int |\nabla_{x}f(x,a^{⋆}){|}_{s}^{q}\;\mu (\text{d}x)\right)}^{(1/q)-1}\cdot \int \frac{\nabla_{x}\nabla_{a}f(x,a^{⋆})\;h(\nabla_{x}f(x,a^{⋆}))}{|\nabla_{x}f(x,a^{⋆}){|}_{s}^{1-q}}\;\mu (\text{d}x),\\ \Delta_{N} & \;:={\left(\int |\nabla_{x}f(x,a^{⋆,N}){|}_{s}^{q}\;\mu_{N}(\text{d}x)\right)}^{(1/q)-1}\cdot {\left(\int \nabla_{a}^{2}f(x,a^{⋆,N})\;\mu_{N}(\text{d}x)\right)}^{-1}\\ & \;\;\cdot \int \frac{\nabla_{x}\nabla_{a}f(x,a^{⋆,N})\;h(\nabla_{x}f(x,a^{⋆,N}))}{|\nabla_{x}f(x,a^{⋆,N}){|}_{s}^{1-q}}\;\mu_{N}(\text{d}x)-{\left(\nabla_{a}^{2}V(0,a^{⋆})\right)}^{-1}\Theta . \end{array}$$

Almost surely (w.r.t. sampling of $\mu_{N}$), we know that $\mu_{N}\to \mu$ in $W_{p}$ as N→∞, and under the regularity and growth assumptions on f in [9, equation (8.2)] we check that $\Delta_{N}\to 0$ a.s., see [9, example 8.4] for details. In particular, taking $\delta =1/\sqrt{N}$ and combining the above with (3.7) we obtain

3.10 $$\sqrt{N}\left(a_{1/\sqrt{N}}^{⋆,N}-a^{⋆}\right)\Rightarrow {\left(\nabla_{a}^{2}V(0,a^{⋆})\right)}^{-1}(H-\Theta ).$$

This recovers the central limit theorem of [47], as discussed in more detail in §4b below. Together, (3.6) and (3.9) give us the a.s. asymptotic behaviour of the out-of-sample error

3.11 $$V(0,a_{\delta }^{⋆,N})-V(0,a^{⋆})=\frac{1}{2N}{\left(H-\Theta \right)}^{T}(\nabla_{a}^{2}V{\left(0,a^{⋆})\right)}^{-1}(H-\Theta )+o\left(\frac{1}{N}\right).$$

These results also extend and complement [48, Prop. 17]. [48] investigate when the distributionally robust optimizers $a_{\delta }^{⋆,N}$ yield, on average, better performance than the simple in-sample optimizer $a^{⋆,N}$. To this end, they consider the expectation, over the realizations of the empirical measure $\mu_{N}$ of

$$V(0,a_{\delta }^{⋆,N})-V(0,a^{⋆,N})=\int f(x,a_{\delta }^{⋆,N})\;\mu (\text{d}x)-\int f(x,a^{⋆,N})\;\mu (\text{d}x).$$

This is closely related to the out-of-sample error and our derivations above can be easily modified. The first-order term in the Taylor expansion no longer vanishes and, instead of (3.6), we now have

$$V(0,a_{\delta }^{⋆,N})-V(0,a^{⋆,N})=\nabla_{a}V(0,a^{⋆,N})(a_{\delta }^{⋆,N}-a^{⋆,N})+o(|a_{\delta }^{⋆,N}-a^{⋆,N}|),$$

which holds, e.g. if for any r>0, there exists c>0 such that $\sum_{i=1}^{k}|\nabla_{a}\nabla_{a_{i}}f(x,a)|\leq c(1+|x{|}^{p})$ for all x∈S, |a|≤r. Combined with (3.8), this gives asymptotics in small δ for a fixed N. For quadratic f and taking q↑∞, we recover the result in [48, Prop. 17], see [9, example 8.4] for details.

## 4. Further discussion and literature review

We start with an overview of related literature and then focus specifically on a comparison of our results with the CLT of [47] mentioned above.

### (a) Discussion of related literature

Let us first remark, that while theorem 2.2 offers some superficial similarities to a classical maximum theorem, which is usually concerned with continuity properties of δ↦V(δ), in this work, we are instead interested in the exact first derivative of the function δ↦V(δ). Indeed, the convergence $\lim_{\delta \to 0}\underset{\nu \in B_{\delta }(\mu )}{\sup}\int f(x)\;\nu (\text{d}x)=\int f(x)\;\mu (\text{d}x)$ follows for all f satisfying $f(x)\leq c(1+|x{|}^{p})$ directly from the definition of convergence in Wasserstein metric (e.g. [49, Def. 6.8]). In conclusion, the main issue is to quantify the rate of this convergence by calculating the first derivative $V^{\prime }(\delta )$.

Our work investigates model uncertainty broadly conceived: it includes errors related to the choice of models from a particular (parametric or not) class of models as well as the mis-specification of such a class altogether (or indeed, its absence). In the decision theoretic literature, these aspects are sometimes referred to as model ambiguity and model mis-specification, respectively, see [50]. However, seeing our main problem (1.2) in decision theoretic terms is not necessarily helpful as we think of f as given and not coming from some latent expected utility type of problem. In particular, our actions a∈A are just constants.

In our work, we decided to capture the uncertainty in the specification of μ using neighbourhoods in the Wasserstein distance. As already mentioned, other choices are possible and have been used in past. Possibly, the most often used alternative is the relative entropy, or the Kullblack–Leibler divergence. In particular, it has been used in this context in economics, see [51]. To the best of our knowledge, the only comparable study of sensitivities with respect to relative entropy balls is [22], see also [45] allowing for additional marginal constraints. However, this only considered the specific case f(x,a)=f(x) where the reward function is independent of the action. Its main result is

$$\underset{\nu \in B_{\delta }^{KL}(\mu )}{\sup}\int f(x)\;\nu (\text{d}x)=\int f(x)\;\mu (\text{d}x)+\sqrt{2{\mathrm{Var}}_{\mu }(f(X))}\delta +\frac{1}{3}\frac{\kappa_{3}(f(X))}{{\mathrm{Var}}_{\mu }(f(X))}\delta^{2}+O(\delta^{3}),$$

where $B_{\delta }^{KL}(\mu )$ is a ball of radius $\delta^{2}$ centred around μ in KL-divergence, ${\mathrm{Var}}_{\mu }(f(X))$ and $\kappa_{3}(f(X))$ denote the variance and kurtosis of f under the measure μ respectively. In particular, the first-order sensitivity involves the function f itself. By contrast, our theorem 2.2 states $V^{\prime }(\delta )={\left(\int {\left(\;f^{\prime }(x)\right)}^{2}\;\mu (\text{d}x)\right)}^{1/2}$ and involves the first derivative $f^{\prime }$. In the trivial case of a point mass $\mu =\delta_{x}$, we recover the intuitive sensitivity $V^{\prime }(\delta )=|f^{\prime }(x)|$, while the results of [22] do not apply for this case. We also note that [22] requires exponential moments of the function f under the baseline measure μ, while we only require polynomial moments. In particular, in applications in econometrics (or any field in which μ typically has fat tails), the scope of application of the corresponding results might then be decisively different. We remark however, that this requirement can be substantially weakened (to the existence of polynomial moments) when replacing KL-divergences by α-divergences, e.g. [52,53]. We expect a sensitivity analysis similar to [22] to hold in this setting. However, to the best of our knowledge no explicit results seem to be available in the literature.

To understand the relative technical difficulties and merits, it is insightful to go into the details of the statements. In fact, in the case of relative entropy and the one-period set-up we are considering, the exact form of the optimizing density can be determined exactly (see [22, Proposition 3.1]) up to a one-dimensional Langrange parameter. This is well known and is the reason behind the usual elegant formulae obtained in this context. But this then reduces the problem in [22] to a one-dimensional problem, which can be well-approximated via a Taylor approximation. By contrast, when we consider balls in the Wasserstein distance, the form of the optimizing measure is not known (apart from some degenerate cases). In fact, a key insight of our results is that the optimizing measure can be approximated by a deterministic shift in the direction ${\left(x+f^{\prime }(x)\delta \right)}_{*}\mu$ (this is, in general, not exact but only true as a first-order approximation). The reason for these contrastive starting points of the analyses is the fact that Wasserstein balls contain a more heterogeneous set of measures, while in the case of relative entropy, exponentiating f will always do the trick. We remark however that this is not true for the finite-horizon problems considered in [22, Section 3.2] any more, where the worst-case measure is found using an elaborate fixed-point equation.

A point which further emphasizes the fact that the topology introduced by the Wasserstein metric is less tractable is the fact that

$$W_{p}^{p}(\mu ,\nu )=\lim_{\varepsilon \to 0}\underset{\pi \in \Pi (\mu ,\nu )}{\inf}\int |x-y{|}^{p}\;\pi (\text{d}x,\text{d}y)+\varepsilon H(\pi |\mu ⊗\nu )=\lim_{\varepsilon \to 0}\varepsilon \underset{\pi \in \Pi (\mu ,\nu )}{\inf}H(\pi |R^{\varepsilon }),$$

where $H(\pi |R^{\varepsilon })=\int \log(\frac{d\pi }{dR^{\varepsilon }})\;d\pi$ is the relative entropy and

$$\text{d}R^{\varepsilon }=c_{0}\exp\left(-\frac{|x-y{|}^{p}}{\varepsilon }\right)\text{d}(\mu ⊗\nu ),$$

for some normalizing constant $c_{0}>0$ (e.g. [54]). This is known as the entropic optimal transport formulation and has received considerable interest in the ML community in the past years (e.g. [55]). In particular, the Wasserstein distance can be approximated by relative entropy, but only with respect to reference measures on the product space. As we consider optimization over ν above it amounts to changing the reference measure. In consequence, the topological structure imposed by Wasserstein distances is more intricate compared to relative entropy, but also more flexible.

The other well-studied distance is the Hellinger distance. [24] calculates influence curves for the minimum Hellinger distance estimator $a^{\mathrm{Hell},⋆}$ on a countable sample space. Their main result is that for the choice f(x,a)=log⁡(ℓ(x,a)) (where ${\left(\ell (x,a)\right)}_{a\in A}$ is a collection of parametric densities)

$$IC(x)=-{\left(\nabla_{a}^{2}V(0,a^{\mathrm{Hell},⋆})\right)}^{-1}\nabla_{a}\log(\ell (x,a^{\mathrm{Hell},⋆})),$$

the product of the inverse Fisher information matrix and the score function, which is the same as for the classical maximum-likelihood estimator. Denote by $\mu_{N}$ the empirical measure of N data samples and by $a^{\mathrm{Hell},⋆}(N)$ the corresponding minimum Hellinger distance estimator for $\mu_{N}$. In particular, the above result then implies the same CLT as for M-estimators given by

$$N^{1/2}(a^{\mathrm{Hell},⋆}(N)-a^{\mathrm{Hell},⋆})\Rightarrow {\left(\nabla_{a}^{2}V(0,a^{\mathrm{Hell},⋆})\right)}^{-1}H,$$

where $H∼N(0,\int \nabla_{a}f{\left(x,a^{\mathrm{Hell},⋆}\right)}^{T}\nabla_{a}f(x,a^{\mathrm{Hell},⋆})\;\mu (\text{d}x)).$ As we discuss in the next section, our theorem 2.4 yields a similar CLT, namely

$$N^{1/2}(a_{1/\sqrt{N}}^{⋆,N}-a^{⋆})\Rightarrow {\left(\nabla_{a}^{2}V(0,a^{⋆})\right)}^{-1}\cdot \left(H-\nabla_{a}\sqrt{\int |\nabla_{x}f(x,a^{⋆}){|}_{s}^{2}\;\mu (\text{d}x)}\;\right).$$

Thus the Wasserstein worst-case approach leads to a shift of the mean of the normal distribution in the direction

$$-\nabla_{a}\sqrt{\int |\nabla_{x}f(x,a^{⋆}){|}_{s}^{2}\;\mu (\text{d}x)},$$

compared to the non-robust case. In the simple case $\mu =N(0,\sigma^{2})$ with standard deviation σ>0, we obtain the MLE $\sigma^{⋆,N}=\frac{1}{N}\sum_{k=1}^{N}X_{i}^{2}$. We can directly compute (for a=σ) that

$$\begin{array}{rl} & \nabla_{\sigma }\sqrt{\int {\left|\nabla_{x}\left(\mathrm{const}.+\log\left(\exp\left(-\frac{x^{2}}{2{\left(\sigma^{⋆}\right)}^{2}}\right)\right)\right)\right|}_{s}^{2}\;\mu (\text{d}x)}=\nabla_{\sigma }\sqrt{\int \frac{x^{2}}{{\left(\sigma^{⋆}\right)}^{4}}\;\mu (\text{d}x)}\\ & \;\;=\nabla_{\sigma }\frac{\sigma^{⋆}}{{\left(\sigma^{⋆}\right)}^{2}}=\nabla_{\sigma }\frac{1}{\sigma^{⋆}}=-\frac{1}{{\left(\sigma^{⋆}\right)}^{2}}. \end{array}$$

Thus the robust approach accounts for a shift of $1/{\left(\sigma^{⋆}\right)}^{2}$ (of order 1 if mulitplied with inverse Fisher information) to account for a possibly higher variance in the underlying data. In particular, in our approach, the so-called neutral spaces considered (e.g. [56], eqn (21)]) as

$$\left\{a:\;-{\left(a-a^{⋆}\right)}^{T}\nabla_{a}^{2}V(0,a^{⋆})(a-a^{⋆})\leq \delta \right\}$$

should also take this shift into account, i.e. their definition should be adjusted to

$$\begin{array}{rl} & \{a:-{\left(a-a^{⋆}+\nabla_{a}\sqrt{\int |\nabla_{x}f(x,a^{⋆}){|}_{s}^{2}\;\mu (\text{d}x)}\right)}^{T}\nabla_{a}^{2}V(0,a^{⋆})\\ & \;\;\cdot \left(a-a^{⋆}+\nabla_{a}\sqrt{\int |\nabla_{x}f(x,a^{⋆}){|}_{s}^{2}\;\mu (\text{d}x)}\right)\leq \delta \}. \end{array}$$

Lastly, let us mention another situation when our approach provides directly interpretable insights in the context of a parametric family of models. Namely, if one considers a family of models P such that the worst-case model in the Wasserstein ball remains in P, i.e. ${\left(x+f^{\prime }(x)\delta \right)}_{*}\mu \in P$, then considering (the first-order approximation to) model uncertainty over Wasserstein balls actually reduces to considerations within the parametric family. While uncommon, such a situation would arise, for example, for a scale-location family P, with μ∈P and a linear/quadratic f.

### (b) Link to the central limit theorem of [47]

As observed in §3e above, theorem 2.4 allows to recover the main results in [47]. We explain this now in detail. Set $||\cdot ||=|\cdot {|}_{s}$, p=q=2, $S=R^{d}$. Let $\mu_{N}$ denote the empirical measure of N i.i.d. samples from μ. We impose the assumptions on μ and f from [47], including Lipschitz continuity of gradients of f and strict convexity. These, in particular, imply that the optimizers $a_{\delta }^{⋆,N},a^{⋆,N}$ and $a^{⋆}$, as defined in §3e are well defined and unique, and further $a_{1/\sqrt{N}}^{⋆,N}\to a^{⋆}$ as N→∞. [47, Thm. 1] implies that, as N→∞,

4.1 $$\sqrt{N}(a_{1/\sqrt{N}}^{⋆,N}-a^{⋆})\Rightarrow {\left(\nabla_{a}^{2}V(0,a^{⋆})\right)}^{-1}\cdot \left(H-\nabla_{a}\sqrt{\int |\nabla_{x}f(x,a^{⋆}){|}_{s}^{2}\;\mu (\text{d}x)}\;\right),$$

where $H∼N(0,\int \nabla_{a}f{\left(x,a^{⋆}\right)}^{T}\nabla_{a}f(x,a^{⋆})\;\mu (\text{d}x)).$ We note that for $||\cdot ||=|\cdot {|}_{s}$ we have

$$h(x)=(\text{sign}(x_{1})\;|x_{1}{|}^{s-1},\ldots ,\text{sign}(x_{k})\;|x_{k}{|}^{s-1})\cdot |x{|}_{s}^{1-s}=\nabla_{x}|x{|}_{s}.$$

Thus

$$\nabla_{a}\sqrt{\int |\nabla_{x}f(x,a^{⋆}){|}_{s}^{2}\;\mu (\text{d}x)}=\frac{\int |\nabla_{x}f(x,a^{⋆}){|}_{s}h(\nabla_{x}f(x,a^{⋆}))\nabla_{x}\nabla_{a}f(x,a^{⋆})\;\mu (\text{d}x)}{\sqrt{\int |\nabla_{x}f(x,a^{⋆}){|}_{s}^{2}\;\mu (\text{d}x)}},$$

and (4.1) agrees with (3.10) which is justified by the Lipschitz growth assumptions on f, $\nabla_{x}f(x,a)$ and $\nabla_{a}\nabla_{x}f(x,a)$ from [47], see [9, equation (8.2)]. In particular, theorem 2.4 implies (4.1) as a special case. While this connection is insightful to establish^2^ it is also worth stressing that the proofs in [47] pass through the dual formulation and are thus substantially different from ours. Furthermore, while theorem 2.4 holds under milder assumptions on f than those in [47], the last argument in our reasoning above requires the stronger assumptions on f. It is thus not clear if our results could help to significantly weaken the assumptions in the central limit theorems of [47].

## 5. Proofs

We consider the case $S=R^{d}$ and ||⋅||=|⋅| here. For the general case and additional details, we refer to [9]. When clear from the context, we do not indicate the space over which we integrate.

**Proof of theorem 2.2.**

For every δ≥0, let $C_{\delta }(\mu )$ denote those $\pi \in P(R^{d}\times R^{d})$ which satisfy

$$\pi_{1}=\mu \;\text{and}\;{\left(\int |x-y{|}^{p}\;\pi (\text{d}x,\text{d}y)\right)}^{1/p}\leq \delta .$$

As the infimum in the definition of $W_{p}(\mu ,\nu )$ is attained (see [49, Theorem 4.1, p. 43]) one has $B_{\delta }(\mu )=\{\pi_{2}:\pi \in C_{\delta }(\mu )\}$.

We start by showing the ‘≤’ inequality in the statement. For any $a^{⋆}\in A_{0}^{⋆},$ one has $V(\delta )\leq \underset{\nu \in B_{\delta }(\mu )}{\sup}\int f(y,a^{⋆})\;\nu (\text{d}y)$ with equality for δ=0. Therefore, differentiating $f(\cdot ,a^{⋆})$ and using both Fubini’s theorem and Hölder’s inequality, we obtain that

$$\begin{array}{rl} V(\delta )-V(0) & \;\leq \underset{\pi \in C_{\delta }(\mu )}{\sup}\int f(y,a^{⋆})-f(x,a^{⋆})\;\pi (\text{d}x,\text{d}y)\\ & \;=\underset{\pi \in C_{\delta }(\mu )}{\sup}\int_{0}^{1}\int \langle \nabla_{x}f(x+t(y-x),a^{⋆}),(y-x)\rangle \;\pi (\text{d}x,dy)\;\text{d}t\\ & \;\leq \delta \underset{\pi \in C_{\delta }(\mu )}{\sup}\int_{0}^{1}{\left(\int |\nabla_{x}f(x+t(y-x),a^{⋆}){|}^{q}\pi (\text{d}x,\text{d}y)\right)}^{1/q}\;\text{d}t. \end{array}$$

Any choice $\pi^{\delta }\in C_{\delta }(\mu )$ converges in p-Wasserstein distance on $P(R^{d}\times R^{d}$) to the pushforward measure of μ under the mapping x↦(x,x), which we denote ${\left[x\mapsto (x,x)\right]}_{*}\mu$. This can be seen by, for example, considering the coupling ${\left[(x,y)\mapsto (x,y,x,x)\right]}_{*}\pi^{\delta }$ between $\pi^{\delta }$ and ${\left[x\mapsto (x,x)\right]}_{*}\mu$. Now note that q=p/(p−1) and the growth assumption on $\nabla_{x}f(\cdot ,a^{⋆})$ implies

5.1 $$|\nabla_{x}f(x+t(y-x),a^{⋆}){|}^{q}\leq c(1+|x{|}^{p}+|y{|}^{p}),$$

for some c>0 and all $x,y\in R^{d}$, t∈[0,1]. In particular, $\int |\nabla_{x}f(x+t(y-x),a^{⋆}){|}^{q}\;\pi^{\delta }(\text{d}x,\text{d}y)\leq C$ for all t∈[0,1] and small δ>0, for another constant C>0. As further $(x,y)\mapsto |\nabla_{x}f(x+t(y-x),a^{⋆}){|}^{q}$ is continuous for every t, the p-Wasserstein convergence of $\pi^{\delta }$ to ${\left[x\mapsto (x,x)\right]}_{*}\mu$ implies that

$$\int |\nabla_{x}f(x+t(y-x),a^{⋆}){|}^{q}\;\pi^{\delta }(\text{d}x,\text{d}y)\to \int |\nabla_{x}f(x,a^{⋆}){|}^{q}\;\mu (\text{d}x),$$

for every t∈[0,1] for δ→0, see [9, lemma 7.13]. Dominated convergence (in t) then yields ‘≤’ in the statement of the theorem.

We turn now to the opposite ‘≥’ inequality. As V(δ)≥V(0) for every δ>0, there is no loss of generality in assuming that the right-hand side is not equal to zero. Now take any, for notational simplicity not relabelled, subsequence of ${\left(\delta \right)}_{\delta >0}$ which attains the liminf in (V(δ)−V(0))/δ and pick $a_{\delta }^{⋆}\in A_{\delta }^{⋆}$. By assumption, for a (again not relabelled) subsequence, one has $a_{\delta }^{⋆}\to a^{⋆}\in A_{0}^{⋆}$. Further note that $V(0)\leq \int f(x,a_{\delta }^{⋆})\;\mu (\text{d}x)$ which implies

$$V(\delta )-V(0)\geq \underset{\pi \in C_{\delta }(\mu )}{\sup}\int f(y,a_{\delta }^{⋆})-f(x,a_{\delta }^{⋆})\;\pi (\text{d}x,\text{d}y).$$

Now define $\pi^{\delta }:={\left[x\mapsto (x,x+\delta T(x))\right]}_{*}\mu$, where

$$T(x):=\frac{\nabla_{x}f(x,a^{⋆})}{|\nabla_{x}f(x,a^{⋆}){|}^{2-q}}{\left(\int |\nabla_{x}f(z,a^{⋆}){|}^{q}\;\mu (\text{d}z)\right)}^{1/q-1}$$

for $x\in R^{d}$ with the convention 0/0=0. Note that the integral is well defined since, as before in (5.1), one has $|\nabla_{x}f(x,a^{⋆}){|}^{q}\leq C(1+|x{|}^{p})$ for some C>0 and the latter is integrable under μ. Using that pq−p=q it further follows that

$$\begin{array}{rl} & \;\int |x-y{|}^{p}\;\pi^{\delta }(\text{d}x,\text{d}y)=\delta^{p}\int |T(x){|}^{p}\;\mu (\text{d}x)\\ & \;\;=\delta^{p}\frac{\int |\nabla_{x}f(x,a^{⋆}){|}^{p+pq-2p}\;\mu (\text{d}x)}{(\int |\nabla_{x}f(z,a^{⋆}){|}^{q}\;\mu (\text{d}z){)}^{p(1-1/q)}}=\delta^{p}. \end{array}$$

In particular, $\pi^{\delta }\in C_{\delta }(\mu )$ and we can use it to estimate from below the supremum over $C_{\delta }(\mu )$ giving

$$\begin{array}{rl} \frac{V(\delta )-V(0)}{\delta } & \;\geq \frac{1}{\delta }\int f(x+\delta T(x),a_{\delta }^{⋆})-f(x,a_{\delta }^{⋆})\;\mu (\text{d}x)\\ & \;=\int_{0}^{1}\int \langle \nabla_{x}f(x+t\delta T(x),a_{\delta }^{⋆}),T(x)\rangle \;\mu (\text{d}x)\;\text{d}t. \end{array}$$

For any t∈[0,1], with δ→0, the inner integral converges to

$$\int \langle \nabla_{x}f(x,a^{⋆}),T(x)\rangle \;\mu (\text{d}x)={\left(\int |\nabla_{x}f(x,a^{⋆}){|}^{q}\;\mu (\text{d}x)\right)}^{1/q}.$$

The last equality follows from the definition of T and a simple calculation. To justify the convergence, first note that $\left\langle \nabla_{x}f(x+t\delta T(x),a_{\delta }^{⋆}),T(x)\rangle \to \langle \nabla_{x}f(x,a^{⋆}),T(x)\right\rangle$ for all $x\in R^{d}$ by continuity of $\nabla_{x}f$ and since $a_{\delta }^{⋆}\to a^{⋆}$. Moreover, as before in (5.1), one has |T(x)|≤c(1+|x|) for some c>0, hence $|\langle \nabla_{x}f(x+t\delta T(x),a^{⋆}),T(x)\rangle |\leq C(1+|x{|}^{p})$ for some C>0 and all t∈[0,1]. The latter is integrable under μ; hence convergence of the integrals follows from the dominated convergence theorem. This concludes the proof.

**Proof of theorem 2.4.**

We first show that

5.2 $$\begin{array}{rl} \lim_{\delta \to 0}\frac{-\nabla_{a_{i}}V(0,a_{\delta }^{⋆})}{\delta } & \;=\int \nabla_{x}\nabla_{a_{i}}f(x,a^{⋆})\frac{\nabla_{x}f(x,a^{⋆})}{|\nabla_{x}f(x,a^{⋆}){|}^{2-q}}\;\mu (\text{d}x)\\ & \;\;\cdot {\left(\int |\nabla_{x}f(x,a^{⋆}){|}^{q}\;\mu (\text{d}x)\right)}^{1/q-1} \end{array}$$

for all i∈{1,…,k}. We start with the ‘≤’ inequality. For any $a\in A^{o}$, we have

$$\nabla_{a}f(y,a)-\nabla_{a}f(x,a)=\int_{0}^{1}\nabla_{x}\nabla_{a}f(x+t(y-x),a)(y-x)\;\text{d}t.$$

Let δ>0 and recall that $a_{\delta }^{⋆}\in A_{\delta }^{⋆}$ converge to $a^{⋆}\in A_{0}^{⋆}$. Let $B_{\delta }^{⋆}(\mu ,a_{\delta }^{⋆})$ denote the set of $\nu \in B_{\delta }(\mu )$ which attain the value: $\int f(x,a_{\delta }^{⋆})\;\nu (\text{d}x)=V(\delta )$. This is non-empty by assumption 2.3 and [9, lemma 7.16]. By [9, lemma 8.5] the function a↦V(δ,a) is (one-sided) directionally differentiable at $a_{\delta }^{⋆}$ for all δ>0 small and thus for all i∈{1,…,k}

$$\underset{\nu \in B_{\delta }^{⋆}(\mu ,a_{\delta }^{⋆})}{\sup}\int \nabla_{a_{i}}f(x,a_{\delta }^{⋆})\;\nu (\text{d}x)\geq 0.$$

Then, using Lagrange multipliers to encode the optimality of $B_{\delta }^{⋆}(\mu ,a_{\delta }^{⋆})$ in $B_{\delta }(\mu )$, we obtain

$$\begin{array}{rl} -\nabla_{a_{i}}V(0,a_{\delta }^{⋆}) & \;\leq \underset{\nu \in B_{\delta }^{⋆}(\mu ,a_{\delta }^{⋆})}{\sup}\int \nabla_{a_{i}}f(y,a_{\delta }^{⋆})\nu (\text{d}y)-\nabla_{a_{i}}V(0,a_{\delta }^{⋆})\\ & \;=\underset{\nu \in B_{\delta }(\mu )}{\sup}\underset{\lambda \in R}{\inf}(\int [\nabla_{a_{i}}f(y,a_{\delta }^{⋆})+\lambda (f(y,a_{\delta }^{⋆})-V(\delta ))]\nu (\text{d}y)\\ & \;\;-\int [\nabla_{a_{i}}f(x,a_{\delta }^{⋆})+\lambda (f(x,a_{\delta }^{⋆})-V(0,a_{\delta }^{⋆}))]\mu (\text{d}x))\\ & \;=\underset{\lambda \in R}{\inf}(\underset{\pi \in C_{\delta }(\mu )}{\sup}\int_{0}^{1}\int \langle \nabla_{x}\nabla_{a_{i}}f(x+t(y-x),a_{\delta }^{⋆})\\ & \;\;+\lambda \nabla_{x}f(x+t(y-x),a_{\delta }^{⋆}),y-x\rangle \;\pi (\text{d}x,\text{d}y)\;\text{d}t\\ & \;\;-\lambda \underset{\pi \in C_{\delta }(\mu )}{\sup}\int_{0}^{1}\int \langle \nabla_{x}f(x+t(y-x),a_{\delta }^{⋆},y-x\rangle \;\pi (\text{d}x,\text{d}y)\;\text{d}t), \end{array}$$

where we used a minimax argument as well as Fubini’s theorem. We note that the functions above satisfy the assumptions of theorem 2.2 for a fixed λ. In particular, using exactly the same arguments as in the proof of theorem 2.2 (i.e. Hölder’s inequality and a specific transport attaining the supremum) we obtain by exchanging the order of lim sup and inf that

5.3 $$\begin{array}{rl} \underset{\delta \to 0}{lim sup}\frac{-\nabla_{a_{i}}V(0,a_{\delta }^{⋆})}{\delta } & \;\leq \underset{\lambda \in R}{\inf}({\left(\int |\nabla_{x}\nabla_{a_{i}}f(x,a^{⋆})+\lambda \nabla_{x}f(x,a^{⋆}){|}^{q}\;\mu (\text{d}x)\right)}^{1/q}\\ & \;\;-\lambda {\left(\int |\nabla_{x}f(x,a^{⋆}){|}^{q}\;\mu (\text{d}x)\right)}^{1/q}). \end{array}$$

For q=2, the infimum can be computed explicitly and equals

$$\frac{\int \langle \nabla_{x}\nabla_{a_{i}}f(x,a^{⋆}),\nabla_{x}f(x,a^{⋆})\rangle \;\mu (\text{d}x)}{\sqrt{\int |\nabla_{x}f(x,a^{⋆}){|}^{2}\;\mu (\text{d}x)}}.$$

For the general case, we refer to [9, lemma 8.6], noting that by assumption $\nabla_{x}f(x,a^{⋆})\neq 0$, we see that the r.h.s. above is equal to the r.h.s. in (5.2).

The proof of the ‘≥’ inequality in (5.2) follows by the very same arguments. Indeed, [9, lemma 8.5] implies that

$$\underset{\nu \in B_{\delta }^{⋆}(\mu ,a_{\delta }^{⋆})}{\inf}\int \nabla_{a_{i}}f(x,a_{\delta }^{⋆})\;\nu (\text{d}x)\leq 0,$$

for all i∈{1,…,k} and we can write

$$\begin{array}{rl} -\nabla_{a_{i}}V(0,a_{\delta }^{⋆}) & \;\geq \underset{\nu \in B_{\delta }^{⋆}(\mu ,a_{\delta }^{⋆})}{\inf}\int \nabla_{a_{i}}f(y,a_{\delta }^{⋆})\;\nu (\text{d}y)-\nabla_{a_{i}}V(0,a_{\delta }^{⋆})\\ & \;=\underset{\nu \in B_{\delta }(\mu )}{\inf}\underset{\lambda \in R}{\sup}(\int [\nabla_{a_{i}}f(y,a_{\delta }^{⋆})+\lambda (f(y,a_{\delta }^{⋆})-V(\delta ))]\nu (\text{d}y)\\ & \;\;-\int [\nabla_{a_{i}}f(x,a_{\delta }^{⋆})+\lambda (f(x,a_{\delta }^{⋆})-V(0,a_{\delta }^{⋆}))]\mu (\text{d}x)). \end{array}$$

From here on, we argue as in the ‘≤’ inequality and conclude that indeed (5.2) holds.

By assumption, the matrix $\nabla_{a}^{2}V(0,a^{⋆})$ is invertible. Therefore, in a small neighbourhood of $a^{⋆}$, the mapping $\nabla_{a}V(0,\cdot )$ is invertible. In particular, $a_{\delta }^{⋆}={\left(\nabla_{a}V(0,\cdot )\right)}^{-1}(\nabla_{a}V(0,a_{\delta }^{⋆}))$ and by the first-order condition $a^{⋆}={\left(\nabla_{a}V(0,\cdot )\right)}^{-1}(0)$. Applying the chain rule and using (5.2) gives

$$\begin{array}{rl} \lim_{\delta \to 0}\frac{a_{\delta }^{⋆}-a^{⋆}}{\delta } & \;={\left(\nabla_{a}^{2}V(0,a^{⋆})\right)}^{-1}\cdot \lim_{\delta \to 0}\frac{\nabla_{a}V(0,a_{\delta }^{⋆})}{\delta }\\ & \;=-{\left(\nabla_{a}^{2}V(0,a^{⋆})\right)}^{-1}{\left(\int |\nabla_{x}f(z,a^{⋆}){|}^{q}\;\mu (\text{d}z)\right)}^{1/q-1}\cdot \int \frac{\nabla_{x}\nabla_{a}f(x,a^{⋆})\nabla_{x}f(x,a^{⋆})}{|\nabla_{x}f(x,a^{⋆}){|}^{2-q}}\;\mu (\text{d}x). \end{array}$$

This completes the proof.

## Footnotes

1 In the case s=1, inspecting the proof, we see that theorem 2.4 still holds since $a^{⋆}$ does not have zero components μ-a.s., which are the only points of discontinuity of h.

2 We thank Jose Blanchet for pointing out the possible link and encouraging us to explore it.

## Data accessibility

The codes used to generate figures in the paper are available on GitHub: http://github.com/JanObloj/Robust-uncertainty-sensitivity-analysis.
